# Neomembrane Formation Suggestive of an Organized Chronic Subdural Hematoma: An Incidental Cadaveric Finding

**DOI:** 10.7759/cureus.114874

**Published:** 2026-08-20

**Authors:** Neha Xalxo, Lalit Ratanpara, Pradip R Chauhan, Sundip Charmode, Rohin Garg, Simmi Mehra

**Affiliations:** 1 Anatomy, All India Institute of Medical Sciences, Rajkot, Rajkot, IND

**Keywords:** angiogenesis, cadaveric dissection, chronic subdural hematoma, fibroblast, neomembrane

## Abstract

Chronic subdural hematoma (cSDH) is an extra-axial lesion characterized by progressive organization and neomembrane formation. Although commonly diagnosed clinically, it may occasionally remain asymptomatic and be identified incidentally. During routine cadaveric dissection for undergraduate teaching, an unusual meningeal finding was observed in the cranial cavity of a 67-year-old male cadaver with no available clinical history. Gross examination revealed two thick, gray-white, well-organized membranous structures over the convexity of the left cerebral hemisphere, deep to the meningeal layer of the dura mater and superficial to the intact arachnoid mater, producing localized compression of the underlying parietal lobe. Histological evaluation demonstrated a multilayered neomembranous architecture with dense fibrocollagenous tissue, fibrovascular proliferation, organizing hematoma, and prominent neovascularization. This cadaveric gross and histological correlation highlights the structural organization of a lesion suggestive of an organized cSDH and may contribute to understanding the structural evolution of cSDH.

## Introduction

Chronic subdural hematoma (cSDH) is an extra-axial collection of blood; however, evidence suggests that it is a pathological process of chronic inflammation, angiogenesis, and progressive neomembrane formation [1,2]. The dura mater, arachnoid mater, and pia mater are the three cranial meninges. The dura mater comprises periosteal and meningeal layers, which separate at specific locations to form endothelial-lined channels called dural venous sinuses [3]. Disruption and irritation of the dural border cell layer induce reparative, angiogenic, and inflammatory responses that lead to the formation of granulation tissue, which subsequently organizes into neomembranes. These membranes develop within the subdural compartment and consist of a thick external membrane and a thinner internal membrane. The external neomembrane contains immature, fragile neocapillaries with increased vascular permeability, contributing to continued exudation and hematoma expansion [4]. cSDHs have a variable anatomical distribution and commonly involve the cerebral convexities, cranial base, and interhemispheric fissure [5]. Histological investigations have revealed varying degrees of membrane maturity, vascularity, and hemorrhagic activity, reflecting distinct stages in lesion evolution [6]. The structural features of membranes formed in cSDH have been classified into four histopathological types based on the degree of connective tissue maturation, inflammatory cell infiltration, vascular proliferation, and hemorrhagic activity [7]. Although cSDH is well documented in clinical and radiological literature, its incidental identification during cadaveric dissection is rarely reported. The present report describes an incidental finding suggestive of an organized cSDH with neomembrane formation, with emphasis on its gross morphological and histopathological characteristics.

## Case presentation

During routine cadaveric dissection for undergraduate teaching in 2026, an incidental unusual finding related to the meninges was observed in the cranial cavity of a 67-year-old male cadaver with no available clinical history. The cadaver was voluntarily donated to the Department of Anatomy, All India Institute of Medical Sciences, Rajkot, Gujarat.

Gross findings

On opening the cranial cavity, the meninges displayed three distinct layers: the dura mater, arachnoid mater, and pia mater. Additionally, there were two thick, gray-white-colored, well-organized intermediate membranous structures present over the convexity of the left cerebral hemisphere in a well-defined area. These were confined in nature and situated between the dura and arachnoid mater on the left side. No evidence of skull fracture, bone erosion, or cranial defect was present, and the inner table of the skull appeared intact.

**Figure 1:**
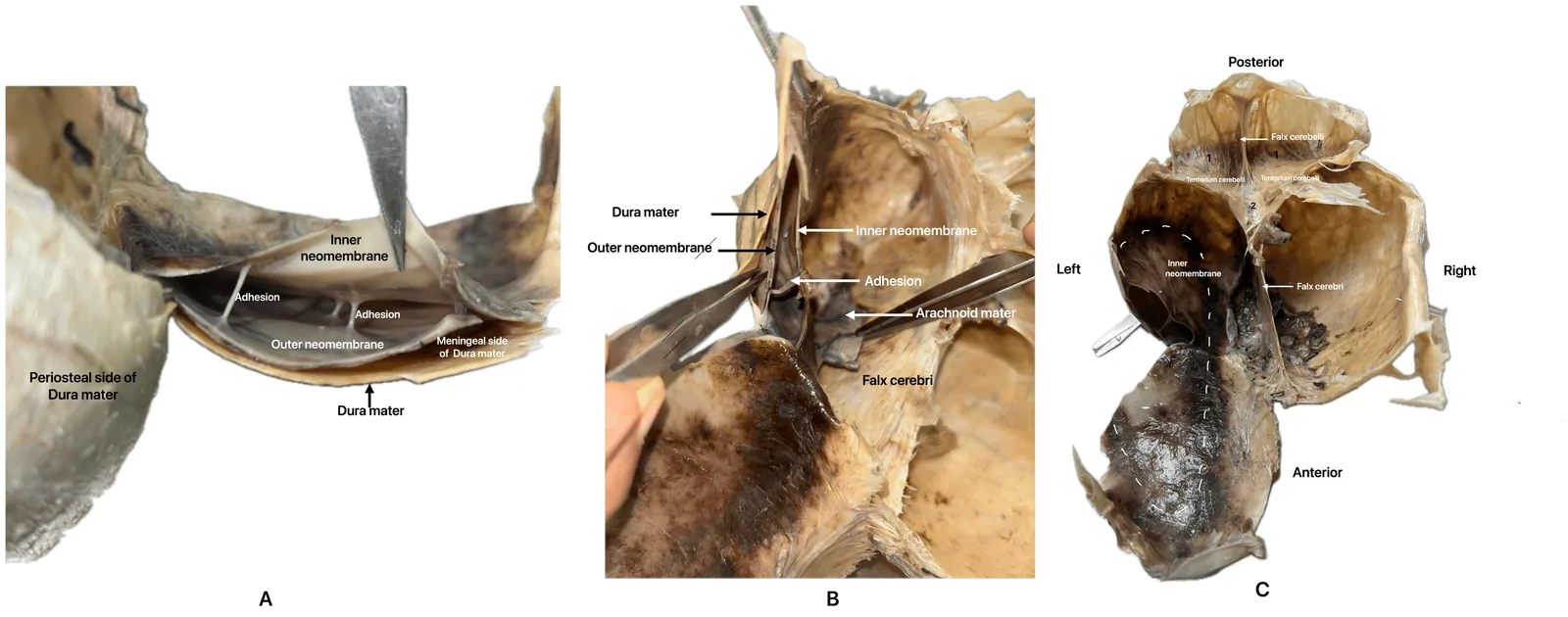
Gross appearance of an organized neomembrane suggestive of chronic subdural hematoma (left side). (A) Gross dissection showing the outer and inner neomembranes with band-like adhesions between them. The periosteal and meningeal sides of the dura mater are shown. (B) Meningeal aspect of the dura mater, showing the outer and inner neomembranes, intervening adhesions, focally detached arachnoid mater, and falx cerebri. (C) Meningeal aspect of the dura mater, depicting the falx cerebri, falx cerebelli, tentorium cerebelli, and inner neomembrane. The area demarcated in white indicates the extent of the organized neomembranous formation. 1: site of the transverse sinus; 2: site of the straight sinus.

One of the membranous structures (outer neomembrane) was located toward the meningeal dura mater and was adherent to it, forming a distinct layer, while the other membranous structure (inner neomembrane) was located toward the arachnoid mater. The two membranes were interconnected by a few band-like adhesions (Figure 1).

**Figure 2:**
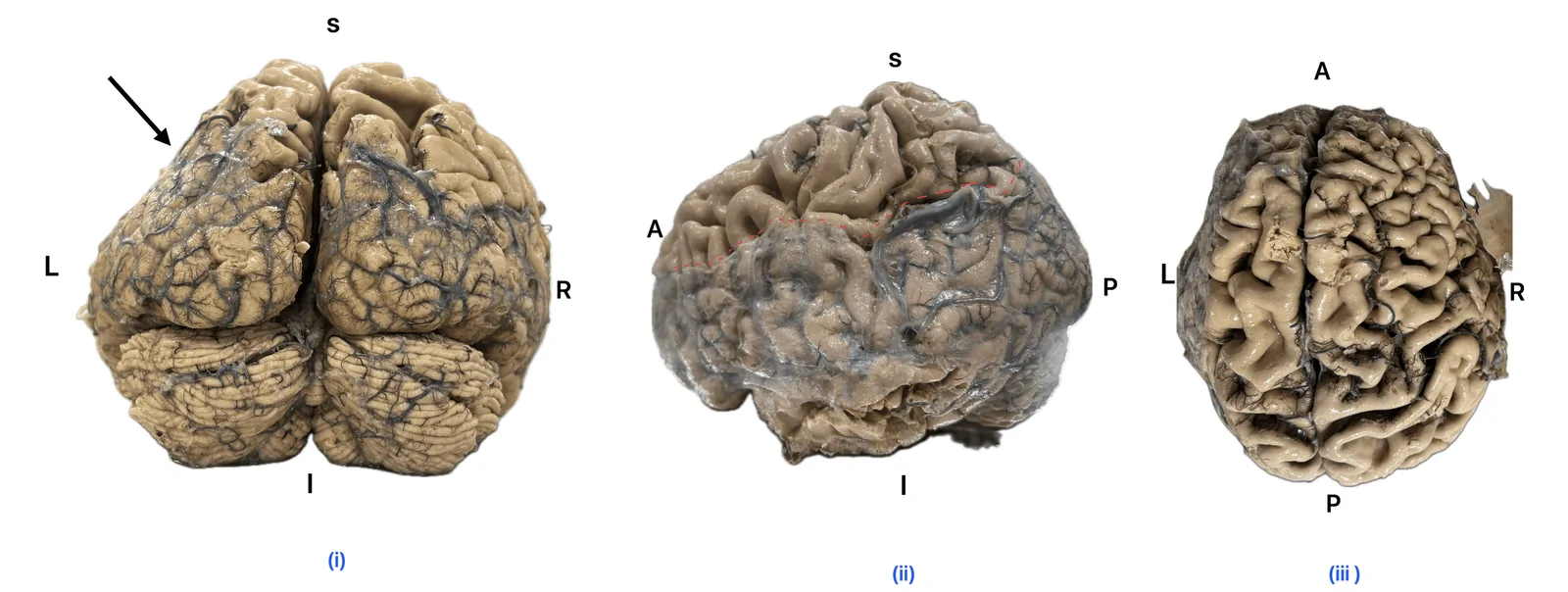
Different aspects of the cerebral hemispheres showing focal depression over the left parietal lobe associated with organized neomembrane formation. (i) Posterior view of the cerebral hemispheres showing focal depression of the left parietal lobe corresponding to the area of the neomembranous lesion. (ii) Lateral view of the left cerebral hemisphere showing the arachnoid mater, which is intact except over the area demarcated by the red dotted line. (iii) Superior view of the cerebral hemispheres. A: anterior; P: posterior; S: superior; I: inferior; L: left; R: right

The arachnoid mater was intact over most of the cerebral hemisphere, with focal areas of detachment from the underlying cerebral surface. It was not adherent to the inner neomembrane and was well separated from the inner neomembranous structure, confirming the extra-axial location. The thickness of the membranous structure close to the dura was greater than that of the membrane located on the arachnoid side. The left cerebral hemisphere over the parietal lobe was depressed and conformed to the shape and size of the neomembrane formation area, suggestive of a pressure effect (Figure 2).

This well-defined area was notably curved, indicating the potential presence of an intermediate layer that might suggest a previous subdural hematoma. However, this could not be conclusively confirmed due to the absence of a medical history, as the cadaver was donated. In contrast, the meninges on the right side were grossly normal. The brain surface on the right side showed no signs suggestive of a hematoma.

Histological findings

**Figure 3:**
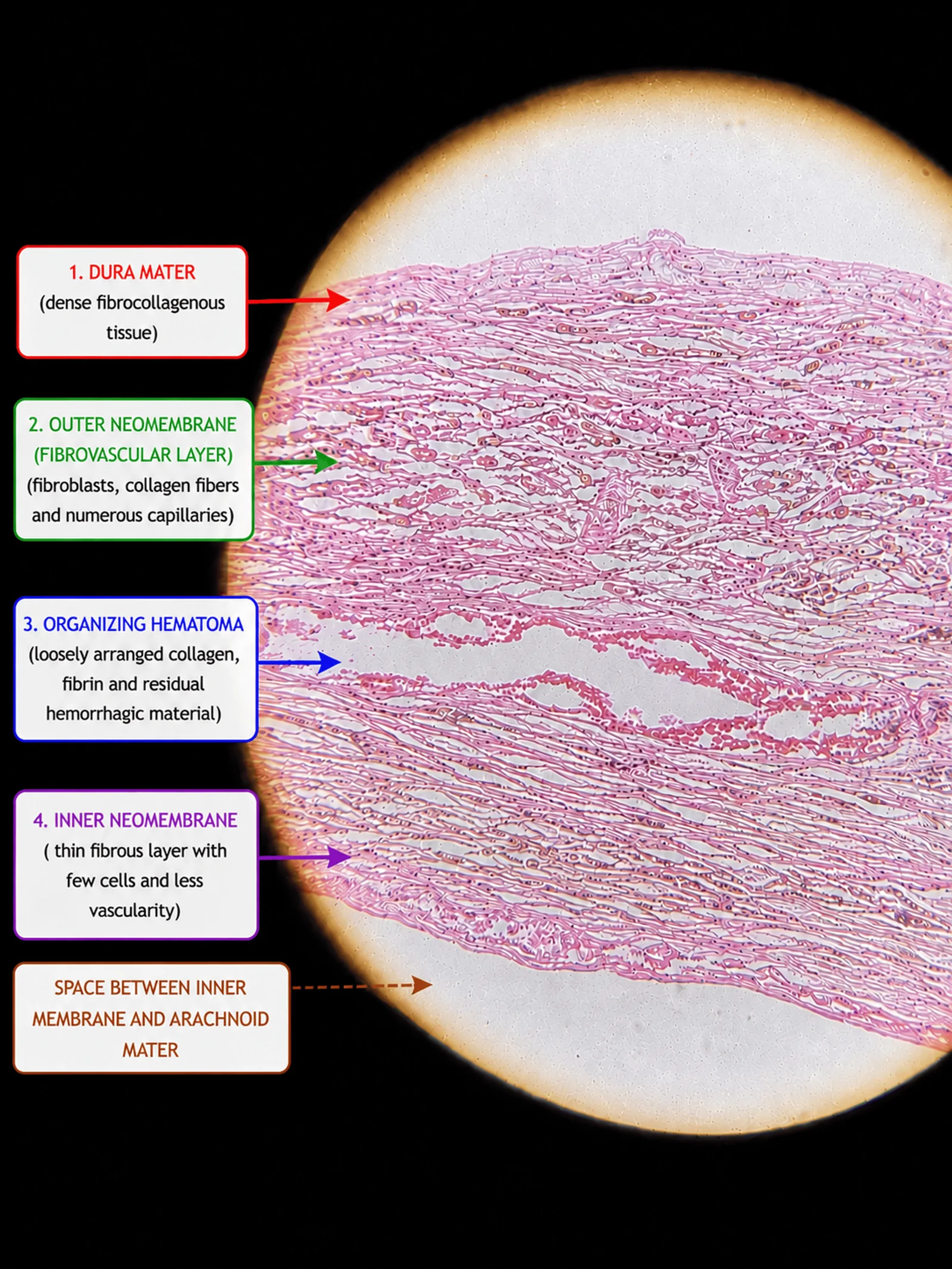
Histological features of suggestive organized chronic subdural hematoma. Histological section showing distinct layers: dura mater, outer fibrovascular neomembrane, organizing hematoma, and inner neomembrane, demonstrating a multilayered organized neomembranous formation.

Histological findings revealed a multilayered extra-axial structure interposed between the dura mater and the arachnoid mater, consistent with the neomembrane formation in an organized cSDH. Distinct histological layers were identified from superficial to deep (Figure 3).

The outermost layer, composed of dense fibrocollagenous tissue, was arranged in thick, parallel bundles. The collagen fibers were compact, with sparse cellularity and minimal vascularity, representing the normal histological architecture of the dura. This layer showed firm continuity with the outer neomembrane, indicating adhesion of the pathological membrane to the inner dural surface.

In the fibrovascular layer (outer neomembrane), immediately beneath the dura mater, there was a thick outer neomembrane, characterized by a fibrovascular architecture. This layer contained numerous fibroblasts, irregularly arranged collagen fibers, and abundant thin-walled capillaries. The high vascularity and cellularity of this layer are typical of the reactive proliferative phase of cSDH and suggest ongoing angiogenesis and inflammatory remodeling. This outer membrane represents the primary source of repeated microhemorrhages described in organized cSDHs.

In the organizing hematoma layer, deep to the outer neomembrane, an intermediate organizing layer was present consisting of loosely arranged collagen fibers with fibrin deposition and residual hemorrhagic material, indicating organization of chronic subdural contents. The heterogeneous appearance of this layer supports the chronicity of the lesion.

In the inner neomembrane, beneath the organizing hematoma, a thinner inner neomembrane was observed. This layer consisted predominantly of compact collagen fibers with low cellularity and minimal to absent vascularity. The reduced vascularity suggests diminished active remodeling at this interface.

The deepest aspect represented the space between the inner neomembrane and the arachnoid mater. This separation is indicative of the extra-axial and subdural location and excludes involvement of the leptomeninges or cerebral cortex. These findings demonstrate a well-organized, multilayered neomembranous formation consistent with an organized cSDH.

## Discussion

It is now believed that many cSDHs develop de novo as a pathological entity, often following trivial or unnoticed head trauma and without progression through a detectable acute subdural hematoma phase [6]. Many patients have no clear history of antecedent trauma. Regardless of the precipitating event, the formation of cSDH is thought to be triggered by disruption and irritation of the dural border cell layer, which constitutes a potential cleavage plane between the dura mater and arachnoid mater. Injury to this layer favors the formation of a fibrovascular neomembrane.

According to the histopathological characteristics, cSDH membranes are categorized into four types [7]. Type I membranes are non-inflammatory with predominant immature fibroblasts and collagen fibers, with little cellular infiltration and few neocapillaries. Type II, called inflammatory membranes, are composed of a single layer of immature connective tissue with prominent infiltration of inflammatory cells and dense vascularization throughout their thickness. Type III membranes are hemorrhagic inflammatory membranes of two or three layers with dilated capillaries on the dural side and numerous thin-walled neovessels on the hematoma cavity side, often with intramembranous hemorrhage. In some cases, an intermediate layer composed mainly of collagen fibers and fibroblasts separates these vascular layers. Type IV membranes represent a scar-forming inflammatory stage, characterized by cicatricial tissue with persistent inflammation, neovascularization, and hemorrhagic changes within the outer membrane [7].

Histological examination of the present case revealed a multilayered fibrovascular membrane with marked neovascularization, significant inflammatory cell infiltration, and features suggestive of intramembranous hemorrhage. These findings most closely correspond to a Type III (hemorrhagic inflammatory) membrane. Furthermore, the observed disparity in thickness between the outer and inner membranous layers, along with fibrous interconnections between them, supports progressive organization and maturation of the lesion. The potential effects of the preservation process on tissue morphology and histological features may represent a limitation when interpreting the present findings. The neovessels within the outer neomembrane are often immature and structurally abnormal, exhibiting endothelial gaps and fenestrations, particularly in patients with neurological deficits [1]. Although clinical correlation was not possible in the present incidental cadaveric finding, the identification of a well-organized hemorrhagic inflammatory membrane associated with focal depression of the underlying cerebral hemisphere suggests that a clinically significant mass effect may have been present during life.

Based on non-contrast computed tomography, the internal architecture of cSDHs has been classified as homogeneous, laminar, separated, and trabecular types, with the separated and trabecular patterns reflecting increased chronicity and membrane organization [8]. While radiological data were unavailable in this cadaveric case, the gross appearance of multilayered membranes with fibrous septations and the corresponding histological features closely resemble the advanced stages described by Nakaguchi et al. [8]. Similar observations have been reported in studies utilizing contrast-enhanced magnetic resonance imaging and dual-energy computed tomography, which demonstrate progressive thickening of the external membrane followed by formation of internal membranes, changes that correlate with lesion chronicity and membrane maturity [9-11]. Cadaveric reports on cSDH are rare. The present case report adds to the limited reports by demonstrating organized neomembrane formation with gross and histological correlation.

## Conclusions

The gross and histological observations in this report demonstrate a well-developed neomembrane suggestive of an organized cSDH; however, the lack of clinical and radiological records limits definitive correlation with clinical history, antecedent trauma, severity, or progression of the lesion. Cadaveric studies provide a unique opportunity to evaluate such untreated lesions in their natural state and allow direct correlation between gross anatomical features and microscopic architecture. Such observations may contribute to a better understanding of the anatomical basis of cSDH evolution. This finding also highlights the potential value of recognizing and documenting the chronic intracranial lesions that may otherwise remain clinically undocumented.
